# Microalgae in Food-Energy-Water Nexus: A Review on Progress of Forward Osmosis Applications

**DOI:** 10.3390/membranes9120166

**Published:** 2019-12-05

**Authors:** Yusuf Wibisono, Wahyunanto Agung Nugroho, Luhur Akbar Devianto, Akhmad Adi Sulianto, Muhammad Roil Bilad

**Affiliations:** 1 Bioprocess Enginering, Brawijaya University, Malang 65141, Indonesia; wahyunanto@ub.ac.id; 2 Environmental Engineering, Brawijaya University, Malang 65141, Indonesia; luhur.devianto@ub.ac.id (L.A.D.); adi_sulianto@ub.ac.id (A.A.S.); 3 Chemical Engineering, Universiti Teknologi Petronas, Bandar Seri Iskandar, Perak 32610, Malaysia; mroil.bilad@utp.edu.my

**Keywords:** microalgae, food-energy-water nexus, osmotic dillution, forward osmosis, membrane

## Abstract

Nowadays the world is facing vulnerability problems related to food, energy and water demands. The challenges in those subsystems are intertwined and thus require inter-discipline approaches to address them. Bioresources offer promising solutions of the dilemma. Microalgae biomass is expected to become a superfood and a favorable energy feedstock and assist in supplying clean water and treat wastewater. Efficient mass production of microalgae, both during upstream and downstream processes, is thus a key process for providing high quality and affordable microalgae biomass. This paper covers recent progress in microalgae harvesting and dewatering by using osmotic driven membrane process, i.e., forward osmosis. Critical factors during forward osmosis process for microalgae harvesting and dewatering are discussed. Finally, perspective on further research directions and implementation scenarios of the forward osmosis are also provided.

## 1. Introduction

Nowadays, many countries around the world have immense concerns about forthcoming uncertainty about the increasing demands of food, energy and clean freshwater. The dilemma of the food-energy-water nexus has generated massive awareness among research communities to develop appropriate technological solutions. The nexus itself is not only defined as the qualitative interactions between the food, energy or water subsystems, but also the quantification links among them as nexus nodes [1]. While looking into the quantitative interaction between the subsystems, water-food linkage nodes contribute to 16% of the demands, water-energy linkage nodes approximately 32%, water-energy-food triangular nodes of 30%, and the rest of 22% directly relates to the climate [2].

Pairwise interactions of the food and the energy systems include the foods used to produce the energy and the energy used in food production. Interrelations between the water and the energy systems consisted of the water used in energy production and the energy used in the water supply. Finally, interconnections between the food and the water systems were the water used in the food production, agriculture impacts water quality and virtual water trade [3]. In order to overcome the challenge, rapid transformations to more resilient food, energy and water security are mandatory, to maintain a circular world economy and sustainability [4].

Biorefineries, among others, provide alternatives to efficiently produce food and feed, green energy and water. The next-generation biorefineries should be highly energy efficient, low in water consumption and at the same time to produce multiple bioproducts such as biochemicals, biomaterials, biofuels, food, feed, pharmaceutical, nutraceuticals, and even store electricity by fixing carbon dioxide into carbon-rich products for energy and chemicals [5]. 

Algal-based biorefineries could be scale-up into industrial-scale for producing biofuels, bioproducts and biopolymers, via biochemical and thermochemical conversions [6,7]. More specifically, fractionation of super biomass (e.g., microalgae, biowaste) could help to address the trilemma [8]. Microalgae have been seen as species which might contribute to the nexus, especially in producing food, being utilized as energy feedstocks or assisting in providing clean water, or even clean air. 

In this paper, the importance of microalgae in food-energy-water nexus and the advances of membrane-based microalgae harvesting and dewatering were scrutinized and reviewed, with the focus on forward osmosis (FO) process. The discussion covers the advantages of microalgae biomass to address the challenge in food-energy-water problems, the factors controlling the efficiency of microalgae harvesting and dewatering using FO membranes, e.g., types and characteristics of the draw solution (DS) and feed solution (FS), types and properties of the FO membranes, and the rates and factors affecting dewatering. After treated in FO process, the biomass filtrate subsequently can be used and processed further to become bioproducts and biofuels. Therefore, the future direction of the research on FO membrane for microalgae harvesting and dewatering are also briefly elaborated.

## 2. Microalgae as a Super Food, Energy Feedstock and Water Cleaner

Currently, the global food sources are predominantly coming from wheat, rice, maize and soybeans, contributed 57% of calorie and 61% of protein requirements. Most of the resources (52%) are, however, contributed by only five countries, i.e., Brazil, China, India, Indonesia and the United States. More than 85% of countries depend on food imports [4]. Microalgae biomass is seen as an innovative functional food source, because it is rich in protein and other valuable substances [9]. Microalgae from saline and fresh water can become sources of nutrients such as fatty acids, carotenoids, steroids, lectins, polysaccharides, amino acids, alginic acids, halogenated compounds and carrageenan [10]. Proteins in microalgae contain essential amino acids at concentrations higher than other sources, e.g., meat and dairy products [11]. Not only proteins, but microalgae also contain a great amount of lipid, vitamins, mineral and pigments [12]. Table 1 summarized the comparable ingredients of microalgae and conventional food source. It shows that microalgae biomass is not only highly in proteins, but certain microalgae species also contain significant carbohydrates and lipids. The rich proteins, carbohydrates, and lipids contents of microalgae (both freshwater and saline microalgae), even somehow higher than that of animal and terrestrial plant sources.

Microalgae can grow rapidly in a very marginal areas, which is the key to implementing microalgae for food and energy sources [14]. Since 1950, when the era of “Great Acceleration” begun, fossil fuel-based energy from coal, oil and gas have been explored and exploited enormously [15]. After seven decades, the massive uses of fossil fuels have reached a critical condition, i.e., highly polluted environment, population explosion, atmospheric destruction, ocean acidification and climate change. Therefore, a more sustainable and renewable energy sources must be urgently invented and implemented to overcome the present energy problem. Such a situation stimulates developments of new and viable bio-based economy in form of exploration of alternative energy sources [16].

The first-generation of bioethanol was mainly derived from cereal and sugar crops, while the first-generation of biodiesel was coming from edible vegetable oils as the feed-stocks. Competition between food and energy utilization arose in this context, and even promotes carbon debt due to changes in the land usage to compensate the need to produce feedstock for bioenergy generation [17]. While utilizing microalgae as food source is definitely reliable, the uses of microalgae for bioenergy feedstock are also promising and have so far been extensively explored. Microalgae biomass is considered as the third-generation of biodiesel and bioethanol feedstock. It is seen as the most sustainable resource for supplying feedstock for biofuels production. 

Lipids in the form of free fatty acids are primarily used as the biodiesel raw materials, while carbohydrates are used as feedstock for the fermentation process to produce bioethanol. The residue of the microalgae biomass, after lipid or polysaccharides extraction, is yet still potentially used as biogas feedstock [13]. Extracted lipid from microalgae could reach 136,900 L/ha area cultivation, >23 times higher than that of oil palm oil, which is the highest among terrestrial crops [18]. Cultivating microalgae in the open-ponds systems however requires precise calculation on the water management and the energy balance [19]. While enclosed photo-bioreactor offers higher lipid yield [20]. 

Lipids from microalgae are converted via a trans-esterification process to produce biodiesel [21]. Fortunately, there is no change in the biodiesel conversion process while using microalgae lipids [22]. On the other hand, the carbohydrate contents can be converted into bioethanol. Unlike the ones from terrestrial crop biomass, microalgae carbohydrate is in starch form that is easily converted into bioethanol [23]. The lignocellulosic materials from the crops harvested from arable land follow complicated conversion routes, particularly for the polysaccharides separation from lignin. The conversion of bioethanol from microalgae-based feedstock can be done traditionally by hydrolysis and fermentation using bacteria and yeast or via dark fermentation process [24].

Biomass residue of extracted microalgae can be converted into biogas via anaerobic digestion process [25]. Combination of extraction of lipid (converted into biodiesel), protein (for functional food) and carbohydrates (as bioethanol precursor), followed by anaerobic digestion process to utilize the biomass residue could enhance the overall efficiency of the microalgae biomass utilization [26]. The anaerobic digestion process consists of hydrolysis and methanogenesis which produce the biogas (mainly consists of CH_4_ as energy sources and CO_2_) and the digestates as byproducts [27]. In a closed-loop scenario, the CO_2_ from the biogas can be returned back to biomass cultivation, and produces the next biomass [20], making the system highly sustainable. 

The utilization of microalgae biomass as the food sources and the energy feedstock promote land saving for agricultural crops production. Following the intensive used of arable land and exploitation for crop production, the excessive use of chemical fertilizers eventually damages the soil and leaches reactive forms of nitrogen and phosphorus compounds into the surface and groundwater, as well as coastal systems [28,29]. Eutrophication phenomena in the freshwater and the coastal water systems are partly caused by the uncontrolled dispersion of those compounds, which promotes impairment of the environment [30,31]. 

Microalgae are also very useful to provide clean water. Microalgae and bacteria can develop mutual interactions in removing organic materials, excess nutrients (e.g., nitrogen and phosphorus compounds), hazardous contaminants, heavy metal compounds and pathogen from water bodies [32,33]. Microalgae are also very effective organisms for CO_2_ capture. Such roles allow the development of an integrated system that promotes clean air and clean water environment [34].

Microalgae therefore have great potential for addressing the challenges in the food-energy-water trilemma, judging from their important roles in the food-energy-water nexus. Microalgae act as promising bioresources to rapidly convert solar into chemical energy in a sustainable pathway, increase food supplies, mitigate greenhouse gases and offer the possibility to develop a novel route for wastewater remediation [35,36,37]. As the world’s population is foreseen to hit 9 billion by 2050, the development of superfoods, a novel generation of biofuels and novel water treatment pathways are very essential, and microalgae are anticipated to play important roles as enabler resources to address those global threats.

## 3. Microalgae Cultivation, Harvesting and Dewatering

Mass production of microalgae is traditionally done in photoautotrophic environments, in the presences of light as energy source and the CO_2_ as carbon source [38]. On the other hand, heterotrophic conditions can help control rapid growth and produce a high yield of valuable compounds [39]. Microalgae are cultivated either in open pond systems or in closed reactor systems. A photobioreactor is used in microalgae cultivation by implementing bubble columns, airlift, tubular, flat-plate and stirred tank reactors [40]. A more comprehensive description of microalgae cultivation methods can be found elsewhere [41].

The major challenges for microalgae utilization occur during the biomass harvesting process. Microalgae broth has a relatively low biomass concentration, while the tiny microalgae cells are also quite difficult to separate from water, having a density of very close to water, and besides the cells normally have a negative surface charge. All of those properties affect the feasibility of limited processes for separation and isolation [42]. 

The first separation technology for microalgae harvesting is flocculation [43]. Flocculation basically eliminates the repulsive force among the microalgae cells due to its negative surface charge. The electrostatic repulsion between the cells is disturbed by the addition of chemicals which also induce Van der Waals forces to attract each other [44]. Instead of using chemicals such as metal salts, flocculation can also be achieved by regulating the pH of the solution (also known as autoflocculation) or by implementing electrocoagulation [45,46]. The use of microorganism-induced flocculation (biofloculants) is also promising, in addition to the more established biopolymers-based flocculants, such as chitosan [47,48,49].

Another method for microalgae harvesting is sedimentation or gravity settling. The key mechanism of the separation in this process is due to an increase of specific gravity of microalgae cells, which then enhances the biomass settling rate [50,51]. Modification of settling position by adapting inclined settling promotes a faster settling velocity and thus separation rate [52]. A more effective way on microalgae separation via gravity settling combines sedimentation with flocculation processes [53,54]. 

Separation of microalgae biomass using centrifugation is only effective on a very small scale, for instance in laboratory works and is only economically feasible for the production of high-value derivatives, i.e., pharmaceuticals. On a greater scale, centrifugation has high energy demands. Improving centrifugation technology is imperative to compensate the high energy requirement [55]. Air flotation is also an alternative for collecting microalgae biomass. Since the microalgae cells are negatively charged, air bubbles do not interact effectively unless a surfactant is added [56]. The use of another type of gas bubble (e.g., hydrogen, ozone) might improve the flotation performance due to specific interaction between the bubbles and microalgae cells [57].

Since the microalgae biomass is separated for further use, such as for food products and energy feedstock, the separation technology should not interfere with the microalgae cells or disrupt the cell morphology. The use of chemicals should be avoided because they could interact with the microalgae cells and affect purity. The use of high pressure or high shear rate also tends to damage the cells. 

**Figure 1:**
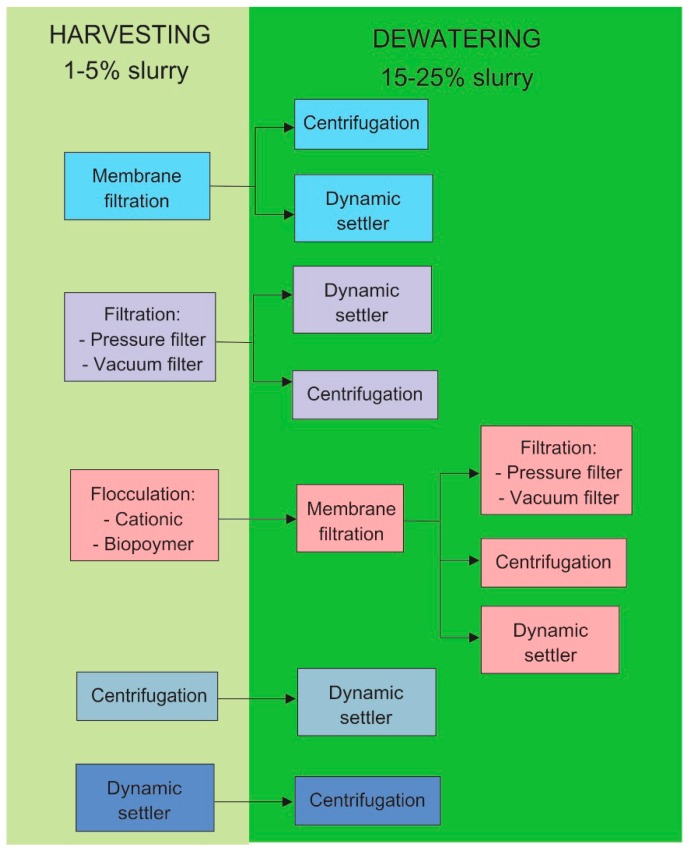
Combination of separation technology for microalgae harvesting and dewatering, adapted and modified from [63].

Filtration-based separation techniques therefore, are seen as a more beneficial for microalgae separation because they employ physical mechanisms. However, due to their mechanical means, the microalgae cell size is the most critical factor. Macrofilters (like filter cloths) and deep filters are only suitable for macroalgae, while membrane filtration is advisable for microalgae separation. Combinations of filtration with other processes, e.g., sedimentation, more likely increase the separation performance [58]. The concentration of microalgae solution is crucial to select appropriate separation technology or combination among them [59]. Microalgae harvesting is concerned with microalgae broth concentrations of 0.05% dry-matter content to a slurry form, typically at 1–5% dry-matter content, while microalgae dewatering increases the concentration of the slurry to an algal cake of 15–25% dry-matter content [42]. Figure 1 shows a schematic diagram of a combination of separation techniques for microalgae harvesting and dewatering.

Membrane-based filtration, can be employed for microalgae harvesting and dewatering (Figure 1). Many membrane-based processes can be utilized: pressure difference driven process, such as microfiltration and ultrafiltration; chemical potential driven process like membrane contactor; and concentration gradient driven process, e.g., dialysis and forward osmosis (FO) [60]. Applications of pressure-driven membrane process for microalgae harvesting and dewatering have been extensively discussed elsewhere [61,62]. Osmotic driven membrane process was barely reported. However, the number of studies involved in this topic is increasing and thus worth for review.

**Figure 2:**
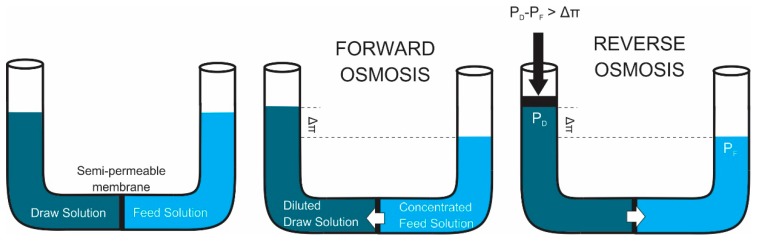
Overview of FO and RO membrane processes (ΔP is hydraulic pressure difference; and Δπ is natural osmotic pressure difference), adapted and modified from [64].

The FO concept has been developed by utilizing the osmotic phenomena. It has been used for separating contaminants and soluble materials from waters through a thin semi-permeable membrane. Despite the fact FO attracted research interest earlier, the process utilizing the reverse stream of water transport known as reverse osmosis (RO) has since developed faster. RO technology reached its milestones earlier, especially for seawater desalination. The RO process however consumes high energy due to the need for the use of high hydrostatic pressure difference. Therefore, the potential of low energy foot-print process in the form of FO has recently extensively been explored. Figure 2 shows the FO and RO processes as a nature and engineered osmotic driven membrane processes.

FO utilizes a concentrated solution to induce concentration gradient between two phases separated by the membrane. The gradient of concentration acts as the driving force for mass-transport in the FO process. The concentrated solution is called as the draw solution (DS), osmotic agent, osmotic media, driving solution, or osmotic engine [65]. As shown in Figure 2, due to concentration gradient between DS and feed solution (FS), water molecules transports from the FS to the DS trough the FO membrane, while solute transports from the DS to the FS [64]. In the RO process, the hydraulic pressure force water transported back to feed solution, when the hydraulic pressure different (ΔP) is larger than the osmotic pressure difference.

## 4. Forward Osmosis for Microalgae Harvesting and Dewatering 

**Figure 3:**
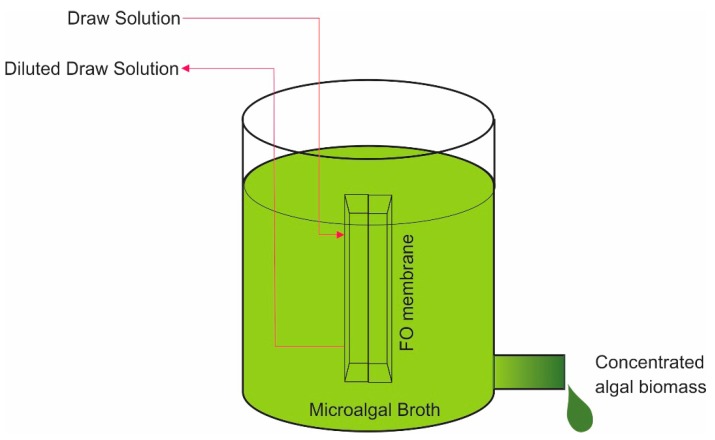
An illustration of a stand-alone FO for microalgae harvesting and dewatering.

An illustration of a stand-alone FO microalgae dewatering process is shown schematically in Figure 3. 

In the stand-alone FO for microalgae harvesting and dewatering, microalgae broth is treated as the FS. High concentration of the DS promotes water molecule diffusion through the FO membrane and increases the concentration of microalgae solution in the feed side. While the DS is continuously flown at the FO membrane active surface, inducing the water transport from the microalgae broth, concentrated algal slurry will eventually be produced.

Studies on the application of FO for microalgae harvesting and dewatering are increasing. The current progresses of the studies are summarized in Table 2. The table shows the comparison among the studies on the use of DS, condition of the FS, the types of FO membrane utilized and the dewatering rate. 

As summarized in Table 2, some aspects can be reviewed to understand the underlying mechanism of microalgae dewatering and harvesting using FO. The involved factors are discussed in Section 4.1, Section 4.2 and Section 4.3.

### 4.1. Draw and Feed Solutions

Primary DS used in FO for microalgae dewatering are synthetic salts, such as NaCl, MgCl_2_, CaCl_2_, KCl, NH_4_Cl [66,67,68,70,71,72,73,74,75,77], mimicking the constituents of seawater. The concentrations used range from 0.5 to 5 M, which is much higher than the salt concentration in seawater [78]. The high salt concentration is intended to promote high osmotic pressure difference, while the feed solution (FS) for marine microalgae such as *Nannochloropsis salina (CCAP 849/3)*, *Piccochlorum sp. BEA0400*, *Porphyridium cruentum* and the hypersaline microalga *Dunaliella salina* BEA0303B which are cultivated using BG-11 and ASP12 medium solutions, contains approximately 0.6 M saline water. When using freshwater microalgae, e.g., *Chlorella vulgaris, Chlamydomonas reinhardtii, Neochloris sp., Choricystis minor* or *Scenedesmus obliquus,* the osmotic pressure difference between DS and FS becomes higher. Both freshwater and saline water microalgae used as FS in the research are dispersed in deionized water to minimize the effect of other factors.

The most explored microalgae were *Chlorella vulgaris*, with different strains from many culture collections, e.g., *Chlorella vulgaris* [67], *Chlorella sp.ADE4* [69], *Chlorella sp. KR-1* [72], *Chlorella vulgaris* (UTEX 2714, Austin, TX) [73], *Chlorella vulgaris* (FACHB-36) [74], *Chlorella vulgaris* KCTC AG 10002 [76]. The microalgae were cultivated with different media such as BG-11, modified bold basal media (BBM), selenite enrichment (SE), and Bristol medium (BM) in different bioreactors. 

Regarding the size and morphology of the microorganisms, they are as follows: *Chlorella vulgaris* is a circular shape microalgae, with a diameter of approximately 2–5 μm, *Chlamydomonas reinhardtii* is also a circular shape microalgae with a bigger diameter of about 10 μm and possesses two flagella, while *Scenedesmus obliquus* has an ellipsoidal shape, with a size of 10 μm (length) and 5 μm (width). The microalgae was cultivated until they reach a density of 1–2 g/L [67] or was resuspended until 23 g/L was reached before use as the FS. 

Natural seawater and sea salts are also used as DS as well as brine solution from brackish water and seawater RO [69,70,71,73]. Pure and crude glycerol was also used as DS [68]. Fresh human urine and hydrolyzed human urine were used as DS, which stimulated up to 3010 kPa osmotic pressure [76], comparable with the osmotic pressure of seawater at 25 °C. Similar results were observed for synthetic urine (both fresh and hydrolyzed) prepared according to previous research [79]. The ammonia contents in urine, however, inhibits microalgae growth. 

### 4.2. FO Membrane Type

Membrane-based cellulose triacetate (CTA) and polyamide thin film composite (TFC) are still dominant polymer manufactured as the FO membranes [80]. Leading manufacturers for FO membranes are Aquaporin, Porifera, HTI and Dow Filmtec. The use of those commercial FO membranes have offered considerably higher water fluxes, especially when coupled with the DS at higher concentrations. Better hydraulic performances are expected when applying more intrinsically superior FO membranes. 

### 4.3. Dewatering Rate

Higher DS concentrations increase the dewatering rate to reach water fluxes of up to 55.4 L/m^2^.h, as shown by several reports summarized in Table 2. Nonetheless, the approach of using highly concentrated DSs shall be carefully considered. The recovery of the DS is one of the main energy consumption factors in FO. The application of concentrated DS can only be attractive when the availability is abundant without the need for recovery, i.e., RO brine used before discharge to the seawater. A more attractive scenario of FO is by the implementation of a stand-alone process when FO is truly energy efficient [64].

## 5. Future Perspectives

Significant progress has been made on the implementation of FO for microalgae harvesting and dewatering. FO has been seen as an attractive process for microalgae biomass separation due to its low energy consumption and its good resistance to membrane fouling. The research so far is focused on the exploration of different membrane types, a variation of DSs and their concentrations for specific microalgae broth as the FS. Most research is still addressing the proof-of-concept aspect and have been mostly done on a lab-scale standalone system. There is no comprehensive study so far reporting the feasibility of FO process in an upgraded scale. Therefore, only limited data are available and seem to be insufficient to fully address the potential of the FO process for microalgae harvesting and dewatering. Some perspectives on the further development of the FO process are presented, focusing on improving the process design and the membrane materials. 

The combination of FO with other processes for microalgae harvesting and dewatering is proposed to enhance the overall process efficiency. Firstly, hybrid FO microalgae dewatering with fertigation processes can be implemented for agriculture systems in areas intruded by seawater. Seawater intrusion is a phenomenon where saline water penetrates into the coastal zone and diffuses into the freshwater aquifer [81]. Seawater intrusion is triggered by sea-level rises and changes in seaward groundwater discharge. Seawater intrusion leads an increase in the salinity of water in irrigation systems and reduces the yield of food crops such as paddy rice and maize [82]. Saline water intrusion increases soil and water salinity, promoting a shortage of grazing land and fodder crops for livestock production, as well as eliminating fresh fish species in the surface water aquifer [83]. At a larger scale, seawater intrusion on agricultural land leads to a change in rice cropping systems and land use patterns [84].

**Figure 4:**
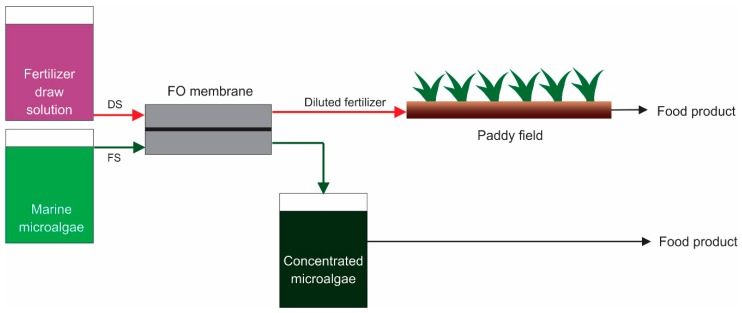
Hybrid process of FO microalgae dewatering and fertigation at saline environment.

Shifts in agricultural practices are proposed to overcome the negative effect of seawater intrusion. The use of salt-tolerable crops, implementing hard-structures to prevent salt diffusion, utilizing advance cultivation techniques and conveying freshwater into agricultural subsystem are appropriate alternatives [85]. Hybrid FO fertigation and microalgae dewatering is proposed as an advanced cultivation technique, as schemed in Figure 4. 

In this hybrid process (Figure 4), marine microalgae such as *Dunaliella salina* or *Nannochloropsis salina* are used as the FS, while conventional fertilizers such as urea, nitrogen and phosphorous compounds are used as the DS. The concentration of fertilizer DS must be higher than the salt concentration in marine microalgae broth to develop a higher osmotic pressure over the FO membrane. Freshwater is transported into fertilizer solution and dilutes the solution prior to use in food crop production. The plants used in the system are the ones for normal freshwater agriculture. The food products are obtained from both harvested food crop and harvested microalgae biomass.

**Figure 5:**
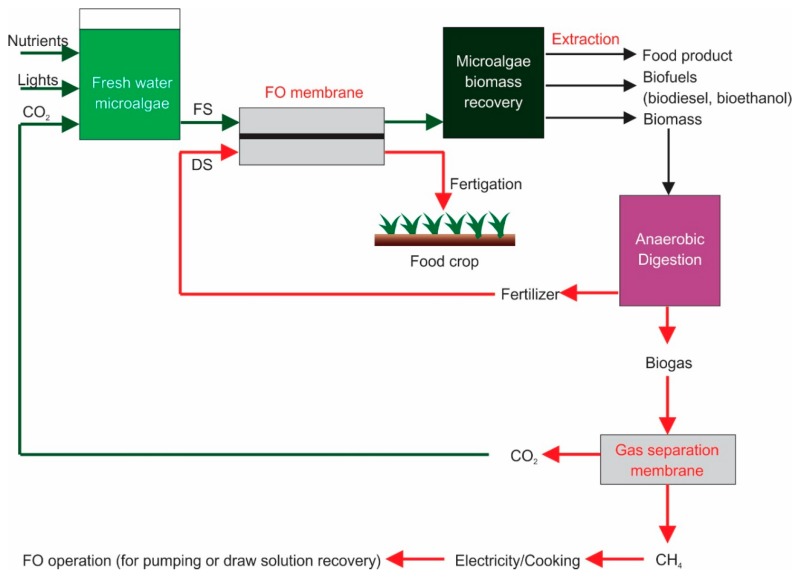
Hybrid process of forward osmosis microalgae dewatering, CO_2_/CH_4_ separation membrane and microalgae-based anaerobic digestion.

Another proposed hybrid process is a combination of FO microalgae dewatering, CO_2_/CH_4_ separation membrane and microalgae-based anaerobic digestion, shown schematically in Figure 5. The system is applied in freshwater microalgae cultures requiring sufficient nutrients, light and CO_2_ supply. The fresh microalgae solution acts as FS, requiring a dewatering process to increase biomass concentration for further treatment, for instance, extraction of valuable compounds or drying of cell biomass. Lipid extract can be converted into biodiesel, carbohydrates can be used directly as food substances or fermented into bioethanol. 

The residual biomass can be used as raw materials for anaerobic digestion processes. The anaerobic digestion produces biogas, which can be treated further using a gas separation membrane to separate CO_2_ and CH_4_. The methane gas can be utilized for electricity production by burning it to drive steam turbines. The produced electricity can be used for FO operations, such as pumping or energy required for DS recovery (especially in hybrid systems). The CO_2_ transferred to the freshwater microalgae feeds provides a carbon source to produce more biomass. The DS solution in FO microalgae dewatering is concentrated into an anaerobic digestion slurry (digestates), which is diluted when is water passed through the FO membrane. The diluted fertilizer is delivered to food agriculture crops to provide nutritional growth. Finally, both harvested food crops and harvested microalgae biomass can be used as food materials, as well as bioenergy resources.

As for FO membrane materials, it is important to switch the materials into sustainable materials and solvents, since the FO is used to treat the bioresources for production of bioproducts and biofuels. Most FO membranes are made from cellulose triacetate and polyamide/polysulfone materials, which traditionally synthesized via phase inversion process use toxic or aggressive organic solvents, e.g., hexane, acetone, dimethylformamide, dimethylsulfoxide, tetrahydrofuran or *n*-methyl-2-pyrrolidone. Organic solvents have a hazardous impact on human health, as well as a major portion of organic pollution is contributed by solvent losses in the environment [86]. The use of non-toxic and non-hazardous solvents are then proposed for manufacturing the FO membranes, such as dihydrolevoglucosenone [87,88,89], methyl lactate [90,91], polyelectrolyte [92,93] or other green solvents [94,95,96]. Although the use of green solvents for the preparation of FO membrane is barely reported, the future development of FO membranes should consider these environment-friendly solvents.

## 6. Concluding Remarks

Microalgae are considered as bioresource materials which are useful for supplying food, energy and clean water. Mass production of microalgae requires advance technology, both for the culturing and harvesting periods. FO membrane processes are recommended to be utilized in microalgae harvesting and dewatering due to its low energy consumption and less fouling tendency. Research on FO for microalgae harvesting and dewatering are predominantly on standalone processes at laboratory scales. Combinations with other processes such as FO fertigation of food crops in saline environments and anaerobic digestion or gas separation membrane are proposed. The hybrid processes are particularly intended to enhance the overall process efficiency for food and energy source production. The use of green solvents for FO membrane fabrication is also proposed, to promote sustainability processes often required when dealing with food, energy and water for future generations.

## Figures and Tables

**Table 1:** Nutritional ingredients of microalgae and other conventional food sources, adapted from [13].

Food Source	Protein	Carbohydrates	Lipids
***Microalgae***			
*Chlorella vulgaris*	51–58	12–17	14–22
*Chlorella pyrenoidosa*	57	26	2
*Chlamydomonas reinhardtii*	48	17	21
*Dunaliella salina*	39–61	14–18	14–20
*Spirulina maxima*	46–63	8–14	4–9
*Spirulina platensis*	52	15	3
***Conventional Food Source***			
Egg	47	4	41
Meat	43	1	34
Soybeans	37	30	20
Milk	26	38	28
Oil palm kernel	16–27	6–11	50–70
Rapeseed	14–18	12–15	40–45

**Table 2:** Concise summary of forward osmosis microalgae harvesting and dewatering.

No.	Feed Solution	Draw Solute and Concentration	Membrane Type	Dewatering Rate		Ref.
				Water Flux (*J_w_*) (L/m^2^·h)	Solute Flux (*J_s_*) (L/m^2^·h)	
1	**Microalgae**: *Chlorella sorokiniana* cultivated in synthetic BG 11, harvested when the concentration reached 2–3 g/L. **FS**: Stock solution diluted with ultrapure water containing 100 mg/L algal biomass.	NaCl (0.5, 1, 2, 4 M) MgCl_2_ (0.5, 2 M)	Flat Sheet CTA FO Membrane embedded with polyester mesh	16.9; 26.8; 36.3; 48.1 22.3; 55.4	0.52; 0.83; 1.17; 1.52 0.13; 0.37	[66]
2	**Microalgae**: *Chlorella vulgaris* cultivated in BG 11 medium and municipal wastewater (as **FS**)	NaCl 35 g/L and natural seawater (35.5 g/L)	Flat Sheet CTA FO Membrane	1.3–2.4	n.a.	[67]
3	**Microalgae**: *Choricystis minor, Scenedesmus sp., Porphyridium cruentum* (from Instituto de Ciencias Marinas, Puerto Real, Spain), *Nannochloropsis salina* (CCAP 849/3) cultivated in different BG 11 mediums added with freshwater or seawater (as **FS**). *Piccochlorum sp*. (BEA0400; Banco Español de Algas, Spain), *Dunaliella salina* (BEA0303B; Banco Español de Algas, Spain) cultivated using ASP 12 Medium (as **FS**).	Pure glycerol Synthetic crude glycerol NaCl (2 M)	Dialysis tubing (Spectrum Labs, USA)	0.63–4.79	n.a.	[68]
4	**Microalgae**: *Chlorella sp.ADE4* was cultivated in BG-11 medium **FS**: Super pure water (FW: 18; JT Baker, USA); Secondary sewage effluent	SWRO concentrate and natural seawater	Flat Sheet CTA FO membrane embedded with polyester screen	2.9 (seawater) 4.8 (SWRO concentrate)	n.a.	[69]
5	**Microalgae**: 0.2 g/L of *Scenedesmus obliquus* biomass in BG-11 medium (as **FS**)	NaCl, MgCl_2_ and CaCl_2_ Commercial sea salt (70 g/L)	CTA and TFC with active layer of polyamide FO membrane, embedded with polyester screen mesh (HTI, USA)	TFC 8.42–8.97 CTA 6.71–9.98	n.a.	[70]
6	**Microalgae**: Freshwater microalgae *Scenedesmus obliquus, Chlamydomonas reinhardtii, and Chlorella vulgaris* Each culture was cultivated in modified BG-11 medium (as **FS**) to reach concentrations of 2–3 g dry weight/L.	Commercial sea salts (70 g/L), MgCl_2_ (86.5 g/L), and CaCl_2_ (114.3 g/L)	Flat Sheet CTA FO membrane supported by an embedded woven mesh (HTI, USA)	Initial flux: 7 Utilizing CaCl_2_ Flux loss: 70.9% (*S. obliquus*), 13.1% (*C. reinhardtii*), 5.3% (*C. vulgaris*) Utilizing Seawater Flux loss: 16.3% (S. *obliquus*), 10.8% (*C. reinhardtii*), 8.1% (*C. vulgaris*)	n.a.	[71]
7	**Microalgae**: *Chlorella sp. (KR-1),* cultivated in nutrient medium **FS**: DI water with initial algal concentration 50 g/L	NaCl (0.5, 1, 2, 5 M)	TFC from Aquaporin Inside^TM^ and Nafion N117 and N211 (DuPont Co. Ltd.)	5–17	n.a.	[72]
8	**Microalgae**: *Chlorella vulgaris* (UTEX 2714, Austin, TX), cultivated in modified bold basal media (BBM) until reach 1 g/L. **FS**: BBM medium with biomass concentration of 1 g/L	NaCl (0.6,1 M) KCl (0.5, 1 M) NH_4_Cl (0.5, 1 M) Natural Seawater (from Florida’s east coast beach)	Flat sheet PES membrane (Aquaporin–Sterlitech, Kent, WA)	5.2 (avg) 7.7 (avg) 6.5 (avg) 7.8 (avg) 4.9 (avg)	0.63 (avg) 0.5 (avg) 1.5 (avg) 0.5 (avg) 0.52(avg) 0.515 (avg)	[73]
9	**Microalgae**: *Chlorella vulgaris* (FACHB-36) cultivated using selenite enrichment (SE) medium until reaching stationary phase (after 25 days) **FS**: Solution from biomass stock filtered from glass fiber membrane (pore size 0.45 µm)	NaCl MgCl_2_ CaCl_2_	TFC membrane (Yantai, China) with active layer of polyamide andsupport layer of polysulfone and CTA membrane with polyester fabric for support layer (HTI, USA)	Water permeability TFC = 1.94 CTA = 0.45	n.a.	[74]
10	**Microalgae**: *Scenedesmus acuminatus* cultured in photobioreactors on modified BG-11 medium **FS**: Deionized water (DI); Re-suspended with microalgal cell in algogenic organic matter (AOM); Algogenic organic matter (AOM); Resuspended microalgal cell in DI water; Original microalgae suspension	MgCl_2_.7H_2_O at 0.5; 1; 2; 3; 4 M	Polyamide TFC-FO membrane (FOMEM-0513, Porifera, USA)	FS-DI Water MgCl_2_ 4 mol: approx 40 MgCl_2_ 4 mol: approx 32 MgCl_2_ 2 mol: approx 26 MgCl_2_ 1 mol: approx 19 MgCl_2_ 0.5 mol: approx 14 FS-1 g/L algae: MgCl_2_ 4 mol: approx 30 MgCl_2_ 4 mol: approx 26 MgCl_2_ 2 mol: approx 24 MgCl_2_ 1 mol: approx 15 FS-23 g/L algae: MgCl_2_ 2–4 mol: approx 13 MgCl_2_ 1 mol: approx 9	n.a.	[75]
11	**Microalgae**: *Chlorella vulgaris,* the Korean Collection for Type Cultures (KCTC) 10002, cultivated in modified Bristol Medium **FS**: DI Water	Fresh urine (Osmotic Pressure: 1340 kPa ) Hydrolyzed urine (Osmotic Pressure: 3010 kPa)	Polyamide TFC (Toray Chemical Korea)	• Real fresh urine: 9.5 (avg) • Synthetic fresh urine: 11 (avg) Real hydrolyzed urine: 16.7 (avg) • Synthetic hydrolyzed urine: 22 (avg)	• Real fresh urine: approx 1.2 TN g/L (Avg) • Synthetic fresh urine: 1.45 TN g/L (avg) • Real hydrolyzed urine: 0.4 TN g/L (avg) Synthetic hydrolyzed urine: 0.15 TN g/L (avg)	[76]
12	**Microalgae**: *Galdieria sulphuraria* cultivated on bioreactor fed with primary effluent wastewater **FS**: Effluent of algae photobioreactor	30 g/L NaCl solution Process: Re-concentration of DS were conducted by using RO unit (SWRO and BWRO)	Hybrid FO—RO Hydrophilic FO membranes (Porifera Inc. California) Aromatic polyamide TFC-RO membranes, brackish water RO (BW30), seawater RO membrane (SW30) (Dow Filmtec, Midland)	0.5–1.5	n.a.	[77]

Noted: approx. (approximately), avg (average), TFC (thin film composite), CTA (cellulose tri acetate), DS (draw solution), FS (feed solution), SW/BW-RO (seawater/brackish water reverse osmosis).
